# Should Public Health Services Be Scrapped?

**Published:** 1921-01-29

**Authors:** 


### THE PUBLIC WELL-BEING.

### SHOULD PUBLIC HEALTH SERVICES BE SCRAPPED?

### Middle Classes and the Doctor's Bill.

The latest criticism of those who are protesting against the logical development of public health schemes in this country is that the latter are actually lowering instead of raising our natural resistance to disease. The argument in support of this sweeping and rather reckless charge seems to be that the means for affording the best medical dietetic and surgical help which used to be found by the middle classes are now lacking; that the middle-classes are the worst sufferers, as they call in a doctor only as the last resort, and cannot afford the expensive little dishes usually prescribed for invalids and children.

This is not the time, therefore, so the argument runs, to introduce fresh schemes for hospitals, sanatoria, and clinics. One would have imagined the conclusions to be the other way about—that at a time when so many of the middle-classes are unable to help themselves it is desirable that more shall be done to give them an opportunity of receiving treatment as adequate and inexpensive as that afforded to the working-classes. It is perfectly true that at the present time doctors' bills, especially in cases of serious illness, form an increasing problem in the lives of the middle-classes—so frequently described now as the new poor—and quite commonly mean the difference between solvency and insolvency. But in our view, based upon a considerable correspondence with people of this class in different parts of the country, the crux of the matter as far as they are concerned lies in the hospital question. It seems a great pity, in some respects, that the system of paying patients was not given a more extended trial on a very much broadened basis, for large numbers of people have been only too quick to express their appreciation of such a scheme, and to show their willingness to be included in it. It is not too much to say that tens of thousands of persons of the middle-class who cannot really afford private treatment in illness, and who do not care to impose on the charitable resources of free treatment at the voluntary hospitals, would welcome the universal adoption of some scheme of carefully graduated hospital payments. It is to be hoped, in the investigations into the general situation of the hospitals on which the Special Committee appointed for the purpose is now embarked, that very careful and sympathetic consideration will be given to this aspect of the problem.

But what is the truth about the cost of the new and still expanding health services that one is given to understand are reducing the nation's physique, and more than any other cause pushing England down the slope towards financial ruin? The gentlemen who scatter statements of that kind upon what they hope will be fruitful soil never mention figures. You must take the figures for granted. Dr. Addison, however, has been supplying the omission, and the result is certainly interesting. He said a day or two ago in public that he recognised that in the call for economy a certain section of the press would seize on the usual object of its attack and cry out against the "squandermaniacs" of the Health Ministry. Recognising in good time that this would happen, he appointed a number of persons to obtain the facts. Briefly, they are said to be as follows: The average increase of rates in 1919 in boroughs examined was 11s. 6d. in the £, a by no means comforting figure; 5s. of that is due to increases in the wages paid by local authorities, 2s. 6½d. to increased cost of material, another 2s. to payments under contracts, and there are a number of additional minor items. All the new health services put together, including construction of roads and houses, extensions of hospitals, sanatoria, services of child welfare, diagnosis and treatment of venereal disease, provision of plant and removal of house refuse and other items, amount to 4¾d. "So that if you save the lot," drily comments the Health Minister, "you will make no perceptible difference." As to the cost to the Exchequer, the total in the Budget for maternity and child-welfare services for England and Wales was £1,100,000—rather less than one-fifth of a penny in the pound.

The physical condition of the next generation is fairly indicated in the health of infancy; and in 1920, after this service began to take effect, the infant death-rate fell to 77, the lowest previously having been 93 in 1917. It would be false economy, adds Dr. Addison, to stop that expenditure, and he is not going to do it. It is not conceivable that the people of Great Britain will ask him to do it. We cannot understand the mentality of such persons as scream for a saving upon one-fifth of a penny in the work of maternity and child welfare. Still less can we understand the intelligence which presents us with the information that saving the child-life of the nation is "lowering the natural resistance" of the British people to disease. It is really an iniquitous statement, which it is the duty of all public-spirited men and women to characterise as it deserves.

